# An Intelligent Cardiopulmonary Training System and Adherence to Training Intensity: A Feasibility Study

**DOI:** 10.3390/ijerph19148335

**Published:** 2022-07-07

**Authors:** Li Wei, Ju-Yang Chi, Jane C.-J. Chao, Yen-Nung Lin

**Affiliations:** 1 Taipei Neuroscience Institute, Taipei Medical University, Taipei 110, Taiwan; weili@tmu.edu.tw; 2 Division of Neurosurgery, Department of Surgery, Wan Fang Hospital, Taipei Medical University, Taipei 110, Taiwan; 3 Graduate Institute of Injury Prevention and Control, Taipei Medical University, Taipei 110, Taiwan; 4 Department of Physical Medicine and Rehabilitation, Wan Fang Hospital, Taipei Medical University, Taipei 110, Taiwan; 110235@w.tmu.edu.tw; 5 School of Nutrition and Health Sciences, College of Nutrition, Taipei Medical University, Taipei 110, Taiwan; chenjui@tmu.edu.tw; 6 Master Program in Global Health and Development, College of Public Health, Taipei Medical University, Taipei 110, Taiwan; 7 Nutrition Research Center, Taipei Medical University Hospital, Taipei Medical University, Taipei 110, Taiwan

**Keywords:** aerobic exercise, artificial intelligence, cardiorespiratory fitness, physical fitness, rehabilitation

## Abstract

**Objective:** Our aim was to evaluate the feasibility of our developed intelligent cardiopulmonary training system (ICTS) and of the percentage of time spent within the target HR range (%time) as an indicator of adherence to training intensity. **Methods:** In this noncontrolled trial, nine participants with sedentary lifestyles were recruited from the outpatient rehabilitation department of a teaching hospital. All participants received twelve 30 min sessions of cycling ergometer exercises (5 min warm up, 20 min training phase, and 5 min cool down) with the ICTS three times per week. Training intensity was determined at 60–80% heart rate reserve using cardiopulmonary exercise (CPET) pretests. During training, pedaling resistance was automatically adjusted by the ICTS to keep the user’s heart rate at the predetermined intensity range. Workload_-peak_ and peak oxygen uptake (VO_2-peak_) were measured during the pretests and post-tests. We recorded the percentage of time spent within the target heart rate range (%time) during the 20 min training phase for each training session as an indicator of adherence. The correlation between %time and gains in VO_2-peak_ was assessed. **Results:** After 4 weeks of training on the ICTS, workload_-peak_ and VO_2-peak_ significantly improved by 13.6 ± 7.2 w (mean ± SD, *p* = 0.008) and 1.5 ± 1.1 mL/kg/min (*p* = 0.011), respectively. The 12-session average %time ranged from 10.6% to 93.1% among the participants, and five participants achieved an average %time >80%. A positive correlation between average %time and training efficacy was found (*rs* = 0.85, *p* = 0.004). **Conclusions:** Cardiopulmonary training with an ICTS is feasible, and the percentage of time spent within the target heart rate range seems to be a reasonable indicator for monitoring training-intensity adherence.

## 1. Introduction

Aerobic exercise is an important component of exercise prescriptions. Aerobic exercise training has been shown to facilitate adaptations of the cardiorespiratory and neuromuscular systems, [1,2,3] such as increasing cardiac output and enhancing oxygen utilization by skeletal muscle [4,5,6]. As a result, it improves aerobic capacity and physical performance [7,8,9]. Higher intensities of aerobic training lead to more favorable outcomes than lower-intensity training [10,11], supporting the clinical importance of sufficient training intensity.

In clinical settings, patients are usually supervised by a physiotherapist (trainer) during aerobic training, and the achievement of target training intensity is often defined as reaching the target heart rate predetermined during a graded cardiopulmonary exercise test (CPET) [12]. If a cycling ergometer is used, both increasing pedaling resistance and cadence are ways of increasing the workload, thereby increasing the patient’s HR. The trainer may adjust the pedaling resistance and/or instruct the patient to adjust cadence to keep their HR within the predetermined level, ensuring that the target intensity is maintained throughout training. However, this method of monitoring is time-consuming and labor-intensive. After transitioning to home- or community-based programs, such a stringent level of personal monitoring will often be unavailable, creating issues of inadequate exercise adherence [13] and lowered training intensity [14,15]. This is especially true in frail or clinical populations, who may have lower self-efficacy compared with healthy populations [13,16]. Workouts that are less intense than intended may lead to undertraining.

To accurately monitor and optimize adherence to training intensity, we introduced a novel system of cardiopulmonary training: the intelligent cardiopulmonary training system (ICTS). Adherence to training intensity was defined as the percentage of time within the target HR range obtained from a CPET test (%time). During training, the pedaling resistance was automatically adjusted by the ICTS to keep the user’s HR within the target HR range. The aim of this study was to investigate the preliminary feasibility of ICTS by evaluating the pre–post aerobic parameters and training adherence of a small group of sedentary subjects.

## 2. Methods

### 2.1. Design

This was a noncontrolled study designed to observe changes in aerobic capacity after 12 sessions (thrice weekly sessions over 4 weeks) of 30 min aerobic training with the ICTS. Adherence to training intensity was determined by measuring the percentage of time a participant kept their HR within the target range during training (i.e., %time), and was recorded every training session. The study was approved by the Joint Institutional Review Board of Taipei Medical University.

### 2.2. Participants

Participants were recruited from the outpatient rehabilitation department of Wan Fang Hospital in Taipei. Subjects aged 20–75 years without regular exercise habits and acute coronary syndrome were eligible to participate in the study. The exclusion criteria were as follows: (1) patients for whom measuring intensity by heart rate reserve (%HRR) may be inaccurate, such as those with arrhythmia, atrial fibrillation, or taking beta-blockers; and (2) those unable to comply with the instructions in CPET and subsequent training.

### 2.3. Cardiopulmonary Exercise Testing

CPET was performed by a rehabilitation specialist in a controlled laboratory. A standardized symptom-limited ramp protocol was performed using a cycle ergometer (VIAsprint 150 P; Ergoline, Bitz, Germany). The participants began with a warm-up phase consisting of a 2 min rest period followed by a 2 min resistance-free pedaling period. The workload was then increased at a ramp rate of 10 or 15 W/min to induce symptom limitation within 8 to 12 min. The participants were asked to maintain a cadence of 50–70 rpm, and the test was terminated if the participant could not maintain the said pedaling rate. Peak capacity was defined as the point at which the participant could no longer tolerate increasing workload with sufficient effort [17]. Each participant breathed through a mask connected to a calibrated volume sensor, which measured the oxygen uptake (VO_2_), carbon dioxide production, and ventilation volume of each breath using a gas analyzer (MasterScreen CPX; CareFusion, Hoechberg, Germany).

### 2.4. Intelligent Cardiopulmonary Training System (ICTS)

The novel ICTS consisted of 4 components: (1) a fitness wristband to monitor the user’s HR; (2) a virtual reality system to provide entertainment; (3) an algorithm to determine pedaling resistance; and (4) a cycling ergometer with 20 levels of pedaling resistance, which was wirelessly adjustable. The ICTS required preset lower and upper limits of the target HR (i.e., 60–80% HRR in this study). At the requested cadence (e.g., 50–70 rpm), the ICTS algorithm determined whether to increase, maintain, or decrease the pedaling resistance, depending on whether the user’s HR was below, within, or above the targeted range, respectively. The goal was to keep the user’s HR within the predetermined range. The algorithm also considered whether the user was pedaling with ease or near exhaustion, based on cadence. For example, when cadence fell below 40 rpm, even if the HR was below the lower limit of target, the system would not increase resistance due to suspected exhaustion.

### 2.5. Interventions

Participants wore a wristband HR monitor and exercised on a cycling ergometer ICTS for 30 min (5 min of warm-up and 20 min of training, followed by 5 min of cooldown). A researcher set up the system and addressed technical issues before each training session, but they did not oversee the training process. Participants were instructed to contact the researcher only if they experienced dizziness, nausea, wheezing, chest pain or palpitations, or any other discomfort that would impede continuation of the session. Participants then performed exercise sessions without supervision. Participants were asked to maintain a cadence of 50–70 rpm throughout the sessions. The target intensity for the 20 min training period was set at 60–80% HRR determined in the CPET pretest using the following formula: HR_-target_ = [(60–80%) × (HR_-peak_ − HR_-rest_)] + HR_-rest_.

### 2.6. Novel Indicator of Adherence

**Figure 1:**
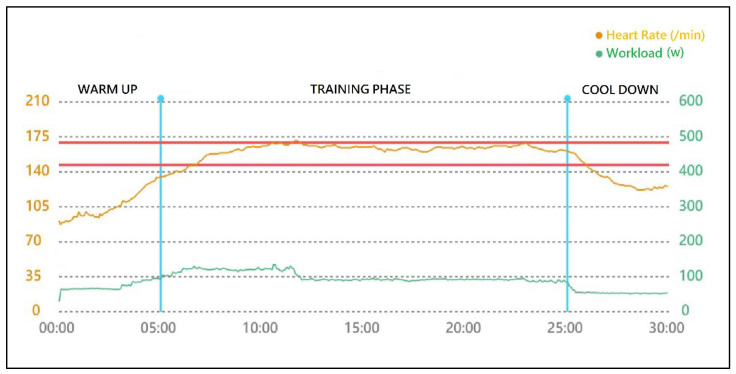
Demonstration of a training-intensity adherence diagram generated by the ICTS. The 2 horizontal red lines indicate the 60% and 80% HRR (147 and 170 bpm, respectively) of this participant according to a predetermined target HR. During the 20 min training phase, the HR was kept within the target range 91.1% of the time.

After training, workload–time and HR–time were plotted. Based on this, we devised an indicator of adherence: the percentage of time within the target HR range (i.e., %time) during the 20 min training phase (Figure 1).

### 2.7. Outcome Measurements

Workload_-peak_ and VO_2-peak_ were obtained by CPET at the pre- and post-test. The indicator of adherence (i.e., %time) was recorded for each training session, and overall adherence was calculated by averaging the %times of the 12 sessions.

### 2.8. Feasibility Indicators

Feasibility indicators were (1) the number of participants retained, (2) the number of sessions attended by participants, and (3) achievement of the planned training intensity (i.e., target heart rate) during training. All adverse events or patient discomfort were recorded.

### 2.9. Statistical Analysis

Wilcoxon matched-pairs signed-rank tests were used to assess pre- and post-test differences. Cohen’s *d_z_* (mean change divided by the standard deviation of change) was calculated to evaluate the effect size of within-group changes. When ES was <0.2, 0.2~0.5, 0.5~0.8, and >0.8, differences were, respectively, found to be trivial, small, moderate, and large [18]. Spearman’s correlation (*rs*) was calculated to evaluate the association between the change in VO_2-peak_ and the indicator of adherence (i.e., %time). Data were analyzed using SPSS version 17.0 (SPSS Inc., Chicago, IL, USA).

## 3. Results

Four men and five women, aged 32–62 years, met the enrollment criteria and completed the training sessions. Individual-level CPET information obtained at the pre- and post-test is summarized in Table 1. At the pretest, the VO_2-peak_ ranged from 25.1 to 33.6 mL/kg/min, and the workload_-peak_ ranged from 63 to 189 w. At the post-test, the VO_2-peak_ and workload_-peak_ changes ranged from −0.1 to 3.9 mL/kg/min and 4 to 30 w_,_ respectively. Significant improvements were noted in workload_-peak_, absolute VO_2-peak_, and relative VO_2-peak_. The effect sizes of within-group changes were large (i.e., Cohen’s *d_z_* > 0.8) for all parameters (Table 2).

**Figure 2:**
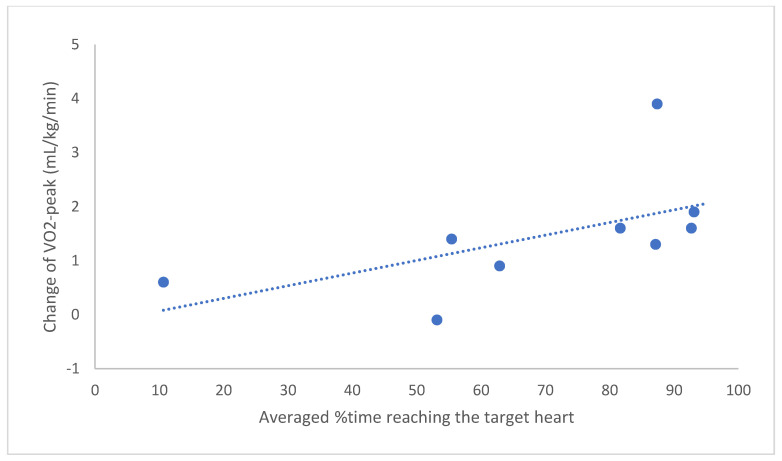
Correlation between adherence to training intensity and the change in aerobic capacity among 9 participants. The *x*-axis indicates the average %time the user’s heart rate was within the target range (60–80% HRR) during the 12 sessions. The *y*-axis indicates the change in VO_2-peak_ between the pre- and post-test (*rs* = 0.85, *p* = 0.004).

The adherence to training in %time is shown in Table 3. The 12-session average ranged from 10.6% to 93.1% among the participants, with an average of 69.9% for the total sample. Eight participants had an average adherence rate > 50%, and five achieved average adherence rates > 80%. No adverse event or patient discomfort was reported. Figure 2 shows a positive correlation between the indicator of adherence (i.e., %time) and improvement in VO_2-peak_ through the training (*rs* = 0.85, *p* = 0.004).

## 4. Discussion

The present study introduced a novel ICTS, which we designed to optimize training adherence. We reported on the feasibility of the ICTS and the proposed indicator of adherence to training intensity. After 4 weeks of training under the ICTS, the workload_-peak_ and VO_2-peak_ were significantly improved among nine sedentary participants, indicating a significant enhancement in aerobic capacity. Overall, the adherence to training intensity was satisfactory, indicating that ICTS use was feasible. We found a positive correlation between the %time spent within the target HR range and the change in VO_2-peak_, suggesting that monitoring %time spent within the target HR range is a potentially useful adherence indicator. Our preliminary results encourage further trials to explore the clinical benefits of applying ICTS in rehabilitation settings and validate the novel adherence indicator.

The ICTS was developed to provide machine-based supervision on a user’s HR during training. In other words, it helps to optimize the user’s adherence to a predetermined training intensity, even without the trainer present. As long as the user pedals within a reasonable cadence range (i.e., 50–70 rpm), the ICTS adjusts pedaling resistance in response to the user’s HR to keep it within the predetermined range. The ICTS may be used in hospital settings, so that supervisors can focus on patient safety without being distracted by the need to adjust training intensity. The automation of intensity adjustment during ergometer cycling training has potential uses in conditions where professional supervision may be less available (e.g., during the COVID-19 pandemic).

In the present study, we found that adherence was acceptable at moderate-to-high intensities (i.e., 60–80% HRR). Eight participants had average %times within the target HR range >50%, and five achieved an average %time >80%. One participant had low adherence, with a %time averaging only 10.6%, possibly due to low motivation (Table 3). This participant refused to increase their cadence above 50 rpm most of the time, despite experiencing minimal exhaustion and no discomfort. Given that the ICTS is unable to discern whether a user has low motivation or is reaching exhaustion, the system did not increase pedaling resistance due to the low cadence rate, despite the patient not reaching their target HR. This result implies that the ICTS may optimize, but does not guarantee, adherence to training. In other words, the system can help screen out subjects with suboptimal adherence. Such information may be useful for clinical practitioners, providing further information about suboptimal adherence. For these subjects, a one-on-one training program may be more suitable.

“Adherence to training” encompasses several dimensions, including adherence to frequency (e.g., how many sessions of exercise per week), intensity (e.g., low, moderate, or vigorous), time (e.g., how many minutes of exercise per session), and type of training (e.g., treadmill or cycling), which is simplified into the acronym FITT. [19] Previous studies addressing adherence to training have usually focused on attendance. [13,20,21]. In comparison, adherence to training intensity has been addressed less often, and there is a lack of generally accepted indicators [22]. Without a proper indicator, it is hard to evaluate whether a trainee receives sufficient training during sessions.

Several indicators of adherence to aerobic-training intensity have been introduced. Cheng et al. [17] recorded HR at the midpoint of each training session, and assessed whether the value reached a predetermined intensity (e.g., 60–80% HRR) in a study with chronic stroke patients. In another study with stroke patients, Gjellesvik et al. [23] measured HR during the last 2 min of the final interval of high-intensity training to evaluate adherence to the training protocol. However, these methods overlooked HR fluctuation. An indicator measuring the total time spent within a predetermined HR range, rather than at a given time point, may provide a more accurate measure of adherence to training intensity. With the advances in technology today, this can be accomplished using wearable devices.

Quevedo-Jerez et al. [24] measured adherence to training intensity by using %time spent in three HR zones (i.e., below the ventilatory threshold, between the ventilatory threshold and respiratory compensation point, and above the respiratory compensation point) during exercise training among cancer survivors. The authors found different patterns of HR-zone distributions in different training modes (i.e., resistance vs. aerobic) and in patients with different aerobic capacities (i.e., <4.5, 4.5–6, or >6 metabolic equivalent). However, they did not evaluate the connection between the clinical outcomes (e.g., VO_2-peak_ gains) and the adherence measurement, giving little clinical insight about the adherence measurement.

In comparison with these studies, we used the %time within the target HR range (i.e., 60–80% HRR, obtained from preintervention CPET) as the indicator of adherence. We explored the correlation between the indicator of adherence and clinical outcomes for the first time. Our finding that patients with better adherence had more favorable gains in VO_2-peak_ support the use of this parameter as a practical indicator of adherence. Further studies using a longer training period (e.g., greater than 4 weeks), with a larger number of respondents, are needed to verify the presented findings.

### Study Limitations

Several limitations should be addressed. First, the sample size was small and without a control group. However, it should be noted that we aimed to report the feasibility rather than the effectiveness of the novel ICTS. Second, as the concept of the ICTS was based on achieving a target HR, this system cannot be recommended for individuals for whom HR measurement would be problematic (e.g., patients using pacemakers or beta-blockers or those with atrial fibrillation). Third, adherence to training may be affected by the level of intensity [13]. The intensity determined in this study was moderate to high; whether the ICTS is useful at higher intensities (e.g., >80% HRR) is unknown.

## 5. Conclusions

Aerobic training with the ICTS is feasible, and the percentage of time spent within the target HR range appears to be a reasonable indicator of adherence to training intensity. Further studies are needed to explore the clinical benefits of applying the ICTS and the role of the novel adherence indicator.

## Figures and Tables

**Table 1:** Basic information and data of cardiopulmonary exercise test of participants.

No.	Sex (M/F)	BMI	Pretest					Post-Test				
		(kg/m^2^)	Workload_-peak_ (W)	VO_2-peak_ (mL/min)	VO_2-peak_ (mL/kg/min)	HR_-peak_ (/min)	RER	Workload_-peak_ (W)	VO_2-peak_ (mL/min)	VO_2-peak_ (mL/kg/min)	HR_-peak_ (/min)	RER
1	F	21.27	123	1520	26.9	173	1.14	146	1633	28.5	165	1.10
2	M	21.75	174	1768	29.6	194	1.16	198	2069	33.5	194	1.24
3	M	30.01	156	2107	25.9	165	1.09	168	2124	26.5	150	1.07
4	F	21.75	114	1510	27.3	163	1.12	133	1522	27.2	169	1.17
5	F	20.55	113	1209	25.1	186	1.20	121	1266	26.7	189	1.21
6	F	21.54	159	1556	28.4	160	1.32	163	1590	29.7	165	1.27
7	M	21.83	189	2175	32.0	189	1.15	196	2301	33.4	177	1.06
8	F	24.00	63	934	18.1	153	1.18	78	987	20.0	163	1.00
9	M	19.76	138	1938	33.6	177	1.05	148	1938	34.5	165	1.05

BMI, body mass index; HR, heart rate; M/F, male/female; RER, respiratory exchange ratio; VO_2_, oxygen uptake.

**Table 2:** Means and SD for pretest, post-test, and changes in aerobic parameters.

	Pretest	Post-Test	Change	Effect Size (*d_z_*)	z-Value	*p*-Value
Workload_-peak_ (w)	136.6 ± 38.2	150.1 ± 37.5	13.6 ± 7.2	1.89	−2.666	0.008
VO_2-peak_ (mL/min)	1628.6 ± 402.9	1714.4 ± 429.7	85.9 ± 89.5	0.96	−2.666	0.008
VO_2-peak_ (mL/kg/min)	27.4 ± 4.5	28.9 ± 4.6	1.5 ± 1.1	1.36	−2.549	0.011

VO_2_, Oxygen uptake.

**Table 3:** The percentage of time (%time) the user’s heart rate was within 60~80% HRR during the 20 min training phase.

No.	S1	S2	S3	S4	S5	S6	S7	S8	S9	S10	S11	S12	Average
1	95.5	93.5	94.4	96.6	99.1	99.3	99.7	99.5	78.3	89.9	80.1	86.6	92.7
2	89.9	77.6	80.3	93.8	92.4	69.8	90.3	89.8	90.9	91.1	93.3	89.5	87.4
3	11.5	9.3	11.8	7.3	5.8	45.8	6.5	2.1	7.6	17.0	1.1	1.8	10.6
4	25.8	76.3	34.5	52.0	71.7	73.5	40.7	12.7	72.3	76.8	47.1	54.3	53.1
5	38.3	62.8	98.8	96.2	100	95.3	96.4	12.9	98.3	99.2	100	X	81.6
6	67.8	62.6	92.0	92.8	89.3	96.6	99.7	92.9	100	88.7	80.3	82.8	87.1
7	78.8	78.5	71.7	62.8	11.4	35.8	56.8	74.7	57.2	63.2	73.17	X	60.4
8	91.9	100.0	96.3	92.0	97.8	93.9	99.1	95.1	88.7	82.0	87.0	X	93.1
9	92.3	54.4	86.7	97.0	58.4	58.1	42.3	71.1	82.9	17.0	32.0	X	62.9

S, sessions; X, the value was not obtainable due to technical problems.

## Data Availability

The data presented in this study are available on request from the corresponding author. Public data sharing is not applicable to this article due to ethical considerations and privacy restrictions.

## Abbreviations

CPET	cardiopulmonary exercise training
HR	heart rate
HRR	heart rate reserve
ICTS	intelligent cardiopulmonary training system
VO_2_	oxygen uptake
